# Supplementary material for: High-Pressure Single-Crystal X‑Ray Diffraction Study of ErVO4
Source: Inorg Chem. 2025 Feb 28;64(10):5202–9. doi: 10.1021/acs.inorgchem.5c00112 (PMC12124714; doi:10.1021/acs.inorgchem.5c00112)
Supplement: Supplementary file 1 [file ic5c00112_si_001.pdf]

Supporting Information

High-pressure single-crystal X-ray diffraction study of $ErVO_4$

Josu Sánchez Martín,[a] Gastón Garbarino,[b] Samuel Gallego-Parra,[b] Alfonso Muñoz,[c] Sushree Sarita Sahoo,[d] Kanchana Venkatakrishnan,[d] Ganapathy Vaitheeswaran,[d] and Daniel Errandonea*[a]

a. Departamento de Física Aplicada-ICMUV, Universidad de Valencia, Dr. Moliner 50, Burjassot, 46100 Valencia, Spain. *E-mail: daniel.errandonea@uv.es

b. European Synchrotron Radiation Facility, Grenoble 38043, France

c. Departamento de Física, MALTA-Consolider Team, Instituto de Materiales y Nanotecnología, Universidad de La Laguna, San Cristóbal de La Laguna, E-38200 Tenerife, Spain

d. School of Physics, University of Hyderabad, Prof. C. R. Rao Road, Gachibowli, Hyderabad, Telangana 500046, India

*Corresponding author; email: daniel.errandonea@uv.es

Table S1. Single crystal XRD refinement data of zircon $ErVO_4$ at selected pressures.

**Pressure (GPa)**	**0.0(1)**	**1.0(1)**	**3.2(1)**	**5.6(1)**	**7.5(1)**
Formula	$ErVO_4$	$ErVO_4$	$ErVO_4$	$ErVO_4$	$ErVO_4$
Dcalc./g·cm^{-3}	5.938	5.977	6.067	6.169	6.238
μ/mm^{-1}	6.793	6.837	6.94	7.056	7.135
Formula Weight	282.2	282.2	282.2	282.2	282.2
Colour	red	red	red	red	red
Shape	plate	plate	plate	plate	plate
Size/μm^3	180×80×20	180×80×20	180×80×20	180×80×20	180×80×20
T/K	293(2)	293(2)	293(2)	293(2)	293(2)
Crystal System	tetragonal	tetragonal	tetragonal	tetragonal	tetragonal
Space Group	$I4_1/amd$	$I4_1/amd$	$I4_1/amd$	$I4_1/amd$	$I4_1/amd$
a/Å	7.09510(10)	7.0770(2)	7.0367(3)	6.9929(2)	6.9641(2)
b/Å	7.09510(10)	7.0770(2)	7.0367(3)	6.9929(2)	6.9641(2)
c/Å	6.2705(2)	6.26150(10)	6.2399(2)	6.2140(2)	6.19600(10)
α/°	90	90	90	90	90
β/°	90	90	90	90	90
γ/°	90	90	90	90	90
V/Å^3	315.660(13)	313.601(18)	308.97(3)	303.87(2)	300.498(18)
Z	4	4	4	4	4
Z'	0.125	0.125	0.125	0.125	0.125
Wavelength/Å	0.41	0.41	0.41	0.41	0.41
Radiation type	Synchrotron	Synchrotron	Synchrotron	Synchrotron	Synchrotron
Θmin/°	2.498	2.501	2.513	2.526	2.537
Θmax/°	20.283	19.879	19.954	20.469	20.097
Measured Refl.	158	177	166	175	166
Indep. Refl.	100	98	91	95	92
Refl. with I >2(I)	96	94	88	93	90
R_{int}	0.019	0.0156	0.0135	0.0156	0.0161
Parameters	12	12	11	11	12
Restraints	0	0	0	0	0
Largest Peak	0.753	0.527	1.204	0.501	0.706
Deepest Hole	-0.581	-1.049	-1.044	-0.726	-0.967
GooF	1.116	1.186	0.613	0.422	0.559
w_{R2} (all data)	0.0492	0.054	0.0646	0.0441	0.0582
w_{R2}	0.049	0.0536	0.0638	0.0441	0.0579
R_1 (all data)	0.0208	0.0204	0.0232	0.0145	0.0183
R_1	0.0207	0.0202	0.023	0.0144	0.0182
CCDC number	2393954	2393950	2393955	2393951	2393952

Table S2. Single crystal XRD refinement data of scheelite $ErVO_4$ at selected pressures.

**Pressure (GPa)**	**7.9(1)**	**10.5(1)**	**12.0(1)**	**14.0(1)**	**19.7(1)**
Formula	$ErVO_4$	$ErVO_4$	$ErVO_4$	$ErVO_4$	$ErVO_4$
Dcalc./g·cm^{-3}	6.988	7.151	7.164	7.231	7.301
μ/mm^{-1}	7.994	8.18	8.196	8.272	8.351
Formula Weight	282.2	282.2	282.2	282.2	282.2
Colour	dark red	dark red	dark red	dark red	dark red
Shape	plate	plate	plate	plate	plate
Size/μm^3	180×80×20	180×80×21	180×80×22	180×80×23	180×80×24
T/K	293(2)	293(2)	293(2)	293(2)	293(2)
Crystal System	tetragonal	tetragonal	tetragonal	tetragonal	tetragonal
Space Group	$I4_1/a$	$I4_1/a$	$I4_1/a$	$I4_1/a$	$I4_1/a$
a/Å	4.9470(10)	4.9074(12)	4.9070(15)	4.8900(10)	4.8780(10)
b/Å	4.9470(10)	4.9074(12)	4.9070(15)	4.8900(10)	4.8780(10)
c/Å	10.960(2)	10.885(3)	10.866(4)	10.840(2)	10.790(2)
α/°	90	90	90	90	90
β/°	90	90	90	90	90
γ/°	90	90	90	90	90
V/Å^3	268.22(12)	262.14(15)	261.63(18)	259.21(12)	256.75(12)
Z	4	4	4	4	4
Z'	0.25	0.25	0.25	0.25	0.25
Wavelength/Å	0.41	0.41	0.41	0.41	0.41
Radiation type	Synchrotron	Synchrotron	Synchrotron	Synchrotron	Synchrotron
Θmin/°	2.600	2.614	2.622	2.628	2.635
Θmax/°	18.269	17.697	17.753	17.947	17.096
Measured Refl.	223	174	183	188	180
Indep. Refl.	122	112	118	116	111
Refl. with I >2(I)	93	86	97	104	89
R_{int}	0.0568	0.0276	0.0333	0.0196	0.0711
Parameters	7	7	9	9	9
Restraints	0	0	0	0	0
Largest Peak	2.893	2.935	4.335	3.68	2.738
Deepest Hole	-2.811	-3.412	-3.439	-3.338	-3.044
GooF	0.986	1.808	2.333	2.432	1.893
w_{R2} (all data)	0.2073	0.2361	0.2926	0.2906	0.3551
w_{R2}	0.1919	0.2272	0.2876	0.2885	0.3472
R_1 (all data)	0.0896	0.0881	0.1061	0.0927	0.1548
R_1	0.0731	0.0754	0.0988	0.0888	0.1419
CCDC number	2393949	2393948	2393953	2393947	2393946

Table S3. Atomic positions determined at selected pressures for zircon $ErVO_4$.

**Pressure (GPa)**		**0.0(1)**	**1.0(1)**	**3.2(1)**	**5.6(1)**	**7.5(1)**
Co	x	0.5	0.5	0.5	0.5	0.5
	y	0.75	0.75	0.75	0.75	0.75
	z	0.875	0.625	0.375	0.625	0.375
V	x	0.5	0.5	0.5	0.5	0.5
	y	0.25	0.75	0.25	0.75	0.25
	z	0.625	0.875	0.125	0.875	0.125
O1	x	0.5	0.5	0.5	0.5	0.5
	y	0.4347(4)	0.5646(6)	0.4355(4)	0.5637(3)	0.4379(4)
	z	0.8003(3)	0.7009(4)	0.3004(3)	0.69965(18)	0.3004(2)

Table S4. Atomic positions determined at selected pressures for scheelite $ErVO_4$.

**Pressure (GPa)**		**7.9(1)**	**10.5(1)**	**12.0(1)**	**14.0(1)**	**19.7(1)**
Co	x	0	0	0	0	0
	y	0.25	0.25	0.25	0.25	0.25
	z	0.625	0.625	0.625	0.625	0.625
V	x	0	0	0	0	0
	y	0.25	0.25	0.25	0.25	0.25
	z	0.125	0.125	0.125	0.125	0.125
O1	x	0.156(3)	0.159(3)	0.160(4)	0.156(3)	0.104(7)
	y	0.509(3)	0.496(4)	0.498(5)	0.506(3)	0.464(8)
	z	0.2078(16)	0.2084(17)	0.212(2)	0.2073(18)	0.215(3)

Table S5. Unit-cell parameters of all measured pressures of zircon (yellow) and scheelite (blue) $ErVO_4$. The “d” mark decompression measurements.

P (GPa)	a, b(Å)	c(Å)	V(Å^3)	P (GPa)	a, b(Å)	c(Å)	V(Å^3)
0.0(1)	7.095	6.271	315.66	11.6(1)	4.912	10.867	262.21
0.4(1)	7.090	6.268	315.11	11.8(1)	4.907	10.863	261.56
0.5(1)	7.088	6.267	314.83	12.0(1)	4.905	10.860	261.23
0.6(1)	7.085	6.266	314.56	12.2(1)	4.899	10.855	260.52
1.0(1)	7.077	6.262	313.60	12.1(1)	4.908	10.857	261.54
1.5(1)	7.069	6.258	312.71	13.2(1)	4.899	10.841	260.21
2.0(1)	7.061	6.253	311.73	13.5(1)	4.897	10.836	259.85
2.5(1)	7.050	6.247	310.47	14.1(1)	4.893	10.824	259.13
3.3(1)	7.037	6.240	308.97	14.8(1)	4.888	10.811	258.29
4.3(1)	7.020	6.231	307.00	15.5(1)	4.884	10.796	257.55
5.1(1)	7.004	6.221	305.13	16.1(1)	4.882	10.784	257.06
5.3(1)	7.000	6.218	304.65	17.2(1)	4.880	10.763	256.31
5.6(1)	6.993	6.214	303.87	18.1(1)	4.873	10.744	255.13
6.5(1)	6.982	6.207	302.53	18.9(1)	4.864	10.731	253.88
7.1(1)	6.967	6.196	300.76	19.7(1)	4.855	10.714	252.57
7.5(1)	6.964	6.196	300.50	20.5(1)	4.849	10.692	251.43
7.9(1)	4.947	10.960	268.22	21.3(1)	4.845	10.675	250.54
8.1(1)	4.953	10.950	268.63	22.0(1)	4.839	10.657	249.52
8.3(1)	4.944	10.945	267.57	22.7(1)	4.835	10.637	248.70
8.6(1)	4.942	10.938	267.13	23.3(1)	4.832	10.622	248.00
9.0(1)	4.938	10.928	266.46	24.2(1)	4.828	10.597	246.99
9.4(1)	4.935	10.920	265.94	19.7(1) d	4.849	10.701	251.61
9.6(1)	4.931	10.912	265.29	15.3(1) d	4.897	10.804	259.08
10.1(1)	4.928	10.903	264.77	11.9(1) d	4.900	10.866	260.92
10.5(1)	4.923	10.891	263.92	9.9(1) d	4.913	10.917	263.50
11.0(1)	4.919	10.872	263.07	7.3(1) d	4.936	10.979	267.46
